# Comparing the Diagnostic Accuracy of the Probe-to-Bone Test, Plain Radiography, and Serum Biomarkers in Detecting Diabetic Foot Osteomyelitis

**DOI:** 10.3390/jcm15020500

**Published:** 2026-01-08

**Authors:** María Herrera-Casamayor, Irene Sanz-Corbalán, Aroa Tardáguila-García, Mateo López-Moral, José Luis Lázaro-Martínez, Yolanda García-Álvarez

**Affiliations:** Diabetic Foot Unit, Clínica Universitaria de Podología, Facultad de Enfermería, Fisioterapia y Podología, Universidad Complutense de Madrid, 28040 Madrid, Spain; maherr14@ucm.es (M.H.-C.); aroa.tardaguila@ucm.es (A.T.-G.); matlopez@ucm.es (M.L.-M.); diabetes@ucm.es (J.L.L.-M.); ygarci01@ucm.es (Y.G.-Á.)

**Keywords:** diabetic foot ulcers, diabetic foot osteomyelitis, probe-to-bone test, plain radiography, erythrocyte sedimentation rate, C-reactive protein

## Abstract

**Background/Objectives:** diabetic foot osteomyelitis (DFO) is a serious complication characterized by bone infection that can involve cortical structures, bone marrow, and surrounding soft tissues. Its prevalence ranges from 20% in moderate diabetic foot infections to over 50% in severe cases, making accurate diagnosis essential in guiding timely and effective management. in this study, we aimed to evaluate the diagnostic accuracy achieved by combining the probe-to-bone (PTB) test, plain radiography, and blood biomarkers—including the erythrocyte sedimentation rate (ESR) and C-reactive protein (CRP)—in the diagnosis of DFO. **Methods:** we conducted a diagnostic accuracy study involving 128 patients with diabetic foot ulcers and clinical suspicion of DFO. The sensitivity, specificity, positive predictive value, and negative predictive value were calculated for individual tests and for their diagnostic combinations. **Results:** the combination of PTB and biomarkers yielded a sensitivity of 75%, a specificity of 24%, a positive predictive value of 69%, and a negative predictive value of 29%. Similarly, the combination of PTB and plain radiography showed a sensitivity of 76%, a specificity of 23%, a positive predictive value of 62%, and a negative predictive value of 38%. When the three diagnostic modalities were analyzed together, the sensitivity reached 75%, and the specificity reached 23%. **Conclusions:** the combination of PTB and inflammatory biomarkers demonstrated moderate effectiveness and diagnostic performance comparable to PTB combined with radiography. These findings suggest that biomarkers may serve as a practical and accessible diagnostic adjunct in settings where imaging availability is limited or radiographic interpretation is challenging.

## 1. Introduction

Diabetic foot osteomyelitis (DFO) is a common and serious complication of diabetic foot disease and remains one of the leading causes of lower-limb amputation. The early and accurate identification of DFO is essential, yet challenging, as clinical presentations are often nonspecific and multiple diagnostic tools are required to establish a definitive diagnosis [1,2,3,4]. It is important to emphasize that diagnosing osteomyelitis requires significant effort from healthcare professionals and a high level of expertise; however, sound clinical practice and the growth of knowledge in this area have led to appropriate treatments, reduced healing times, and, most importantly, a significant decrease in the various complications that may arise, including minor and major amputations. Ensuring timely and accurate diagnosis not only improves patient clinical outcomes but also optimizes resource utilization within the healthcare system, highlighting the critical importance of combining clinical judgment with complementary diagnostic methods in the management of diabetic foot infections.

Several modalities are currently used to evaluate suspected DFO, including plain radiography, the probe-to-bone (PTB) test, magnetic resonance imaging (MRI), bone culture, and bone biopsy [5,6]. While bone biopsy with histology remains the gold standard, it is not routinely performed in clinical practice, particularly when surgical intervention is not indicated [7].

Consequently, less invasive approaches—such as the PTB test, imaging methods, and inflammatory biomarkers—are widely employed to support the diagnosis of osteomyelitis in patients with diabetic foot infections [6,8]. The PTB test is defined as the transulcerous palpation of bone using a blunt, sterile object; it is considered an easy-to-perform, accessible, and low-cost test. Plain radiography provides an overall assessment of osteoarticular structures and offers information about bone involvement, foreign bodies, and soft tissue abnormalities. This test is considered the fastest, most accessible, least expensive, and most easily interpretable imaging method. The PTB test is one of the most frequently used bedside tools, and when combined with plain radiography, it has shown reasonably high diagnostic accuracy. However, radiographic changes typically appear only after the second to fourth week of infection onset, reducing its usefulness in early disease [9,10,11,12].

Inflammatory biomarkers represent another valuable diagnostic adjunct. Infection causes various metabolic alterations that lead to changes in glucose levels. Blood tests can provide suggestive data for osteomyelitis when there is clinical suspicion. The erythrocyte sedimentation rate (ESR), C-reactive protein (CRP), and procalcitonin (PCT) are the most frequently studied serum markers. ESR is a slow-response indicator that is generally higher in DFO than in soft-tissue infections [2,13,14]. PCT may rise earlier—within 4 to 12 h of infection onset—and has been suggested as a potential marker for osteomyelitis [15]. CRP, an acute-phase reactant, tends to decrease sooner during treatment, while ESR may remain elevated for longer, and values above 3.2 mg/dL have been associated with infection [16]. According to the IDSA guidelines [3], decreasing biomarker levels after elevation can be interpreted as a positive prognostic sign.

Previous studies have shown that combining PTB with plain radiography improves diagnostic performance compared with MRI in certain contexts. However, no previous research has assessed the diagnostic accuracy of combining PTB with blood biomarkers in the diagnosis of DFO. This knowledge gap highlights the need to evaluate whether serum markers can enhance accessibility and diagnostic reliability in routine clinical practice.

## 2. Materials and Methods

### 2.1. Study Design

We conducted a diagnostic accuracy study including 128 patients with clinical suspicion of DFO, recruited between May 2023 and July 2025 from a specialized diabetic foot unit.

The inclusion criteria were as follows: adults (≥18 years) with diabetes; clinical suspicion of DFO based on a positive PTB test and/or radiographic findings consistent with osteomyelitis; and the availability of recent laboratory tests.

The exclusion criteria included incomplete medical records; the absence of blood parameter data or laboratory results obtained more than seven days after initial assessment; renal failure; critical limb ischemia; pregnancy or breastfeeding; and chronic conditions such as inflammatory autoimmune disorders (e.g., rheumatoid arthritis, systemic lupus erythematosus). Patients with chronic inflammatory or neoplastic conditions known to significantly affect systemic inflammatory markers were excluded to minimize potential confounding.

### 2.2. Procedure

The study protocol included demographic data collection, vascular and neurological assessment, ulcer evaluation, laboratory testing, PTB testing, plain radiography, and bone culture.

For the assessment of diabetic neuropathy, patients underwent the Semmes–Weinstein 5.07/10 g monofilament test of pressure perception. Three points were evaluated: the pulp of the hallux and the first and fifth metatarsal heads. Neuropathy was considered present if the patient failed to perceive the monofilament at two or more of these locations. Additionally, the vibration perception with a tuning fork was performed on the first and fifth metatarsal heads and the interphalangeal joint of the hallux, with neuropathy considered to be present if the patient failed to perceive vibration at any of these sites [17,18].

Peripheral arterial disease (PAD) was diagnosed based on the absence of both distal pulses (dorsalis pedis and posterior tibial) and the measurement of the ankle–brachial index (ABI) and toe–brachial index (TBI). Peripheral arterial disease (PAD) was diagnosed based on the absence of both distal pulses (dorsalis pedis and posterior tibial). Pulses of the dorsalis pedis and posterior tibial arteries were palpated, and the systolic pressure of both arteries was measured. The ABI was calculated by dividing the highest systolic pressure at the ankles by the highest systolic pressure at the arms, with values <0.9 or >1.4 considered abnormal. The TBI was calculated similarly, using the systolic pressure of the hallux, with values <0.7 considered abnormal [19].

To determine serum biomarker levels, blood samples were collected at the time of clinical suspicion of DFO. Laboratory testing included complete blood counts, routine biochemistry, and specific inflammatory parameters. An elevated erythrocyte sedimentation rate (ESR) was defined as >20 mm/h, elevated procalcitonin (PCT) as ≥0.2 ng/mL, and elevated C-reactive protein (CRP) as ≥3.2 mg/dL [12,14]. To assess the diagnostic accuracy, serum biomarkers were analyzed as a single diagnostic category. A biomarker result was considered positive when at least two of the following parameters exceeded the predefined thresholds: ESR, CRP, or PCT. This approach was used to evaluate the overall diagnostic performance of serum inflammatory markers as an adjunct to clinical assessment and bedside tests [20].

Suspicion of DFO was based on a combination of the PTB test and imaging findings. The PTB test was performed using metal forceps (Halsted mosquito forceps) and was considered positive when the examiner detected a hard or gritty surface [21].

Plain radiographs were classified as positive for osteomyelitis when any of the following features were present: cortical disruption; periosteal elevation; benign or aggressive periosteal reactions; sequestrum formation (devitalized, radiodense bone separated from viable tissue); involucrum (new bone growth surrounding existing bone due to periosteal elevation); or cloacae (cortical openings that allow the discharge of sequestra or granulation tissue).

Bone samples were collected with a strict aseptic technique to prevent contamination from skin flora or adjacent infected tissues. Specimens were transported to the microbiology laboratory following standardized protocols. Pathology samples were fixed in 10% buffered formalin and submitted with patient identification data, including name, medical record number, and anatomical site of infection. All specimens were evaluated by the same pathologist and fixed for 24–48 h.

After fixation, samples underwent decalcification in a softening agent for 2–7 days, depending on bone size and density. Tissues were trimmed to a maximum of 5 mm thickness and 2 cm diameter, embedded in paraffin, and sectioned using a microtome. Sections were stained with hematoxylin and eosin and examined under light microscopy. Laboratory personnel were blinded to all clinical data.

The probe-to-bone test and plain radiography were performed at the time of clinical suspicion of DFO during the initial assessment. Serum biomarker analyses were considered valid only if obtained within seven days of this evaluation. Patients with laboratory results obtained beyond this time frame were excluded to minimize potential temporal bias.

The diagnosis of diabetic foot osteomyelitis was established using a bone biopsy with histological and microbiological analysis in patients who underwent surgical treatment, which is considered the reference standard. In patients managed conservatively, bone culture or deep tissue culture obtained from the ulcer base was used as the reference standard. Therefore, bone biopsy with histological and microbiological analysis, when available, or bone/deep tissue culture in conservatively treated patients, was used as the reference standard and served as the benchmark against which all diagnostic modalities and combinations were evaluated [22,23].

Ethical approval was obtained from the Research Ethics Committee of Hospital Clínico San Carlos (23/144-E), and informed consent was obtained from all participants. The study was conducted in accordance with the Declaration of Helsinki [24].

Regarding treatment, patients without signs of infection received local wound care and offloading. Those with DFUs complicated by soft tissue infection or osteomyelitis received antibiotic therapy tailored to their lesions’ characteristics and their comorbidities. Surgical management was performed in cases where the bone was nonviable or unlikely to heal with conservative treatment.

### 2.3. Statistical Analysis

Data were collected and processed using the IBM SPSS^®^ version 29.0 (SPSS Inc., Chicago, IL, USA). Epidat v4.2 was used to assess diagnostic accuracy. Descriptive analyses were performed, with means and standard deviations calculated for quantitative variables and frequency distributions and percentages calculated for qualitative variables. The χ^2^ test was applied to identify differences in qualitative variables. Odds ratios and 95% confidence intervals were estimated using a univariate model. The McNemar test was used to analyze correlated proportions in paired samples. A *p*-value < 0.05 was considered statistically significant.

To evaluate the primary outcome, receiver operating characteristic (ROC) curves were generated to graphically represent the sensitivity and specificity of each diagnostic test. Additionally, the sensitivity, specificity, positive predictive value (PPV), negative predictive value (NPV), and likelihood ratios (LRs) were calculated for each diagnostic combination.

The sample size was calculated using GRANMO^®^ software version 8.0 (REGICOR, IMIM, Barcelona, SPAIN). A minimum of 128 patients were required to achieve 80% statistical power with a type I error of 5%.

### 2.4. Ethical Principles

This study was conducted in accordance with the standards of the responsible ethics committee (23/144-E), i.e., the Research Ethics Committee of Hospital Clínico San Carlos, which approved it. The authors declare that they have complied with the code of ethics of the Declaration of Helsinki [24]. Prior to inclusion, participants received detailed information about the study and its procedures. Subsequently, they expressed voluntary agreement to participate and gave informed consent for their data to be collected for scientific purposes. To uphold data confidentiality, the data collection notebook maintained patients’ anonymity, and the study results were securely stored in dedicated files created specifically for this purpose, following the security measures mandated by current legislation in Spain.

## 3. Results

A total of 128 patients were included in this study. Descriptive variables are shown in Table 1, and outcome variables are presented in Table 2.

**Figure 1:**
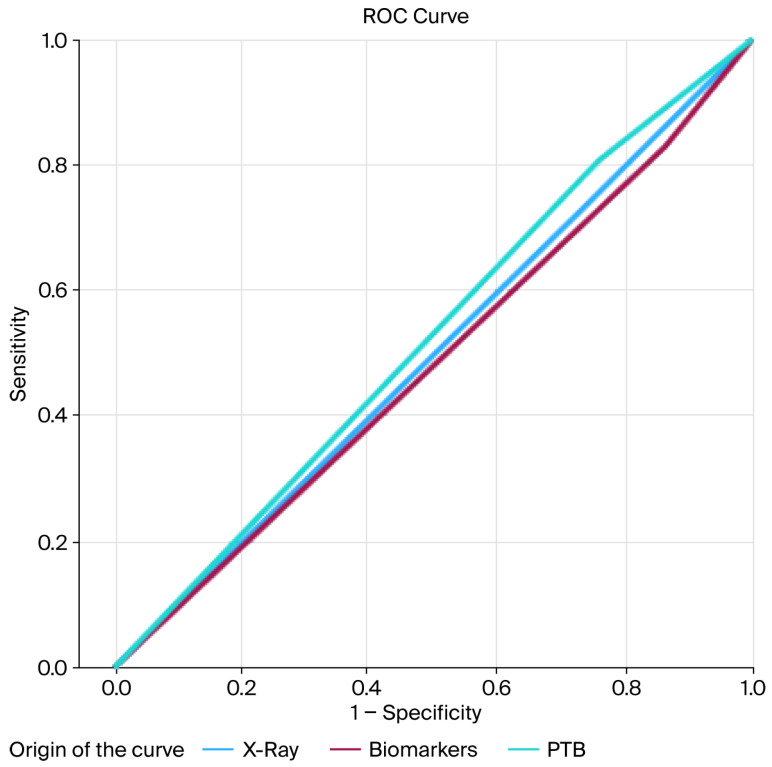
ROC curve of X-ray, PTB, and biomarkers. Abbreviations: X-ray (plain radiography). PTB (probe-to-bone).

**Figure 2:**
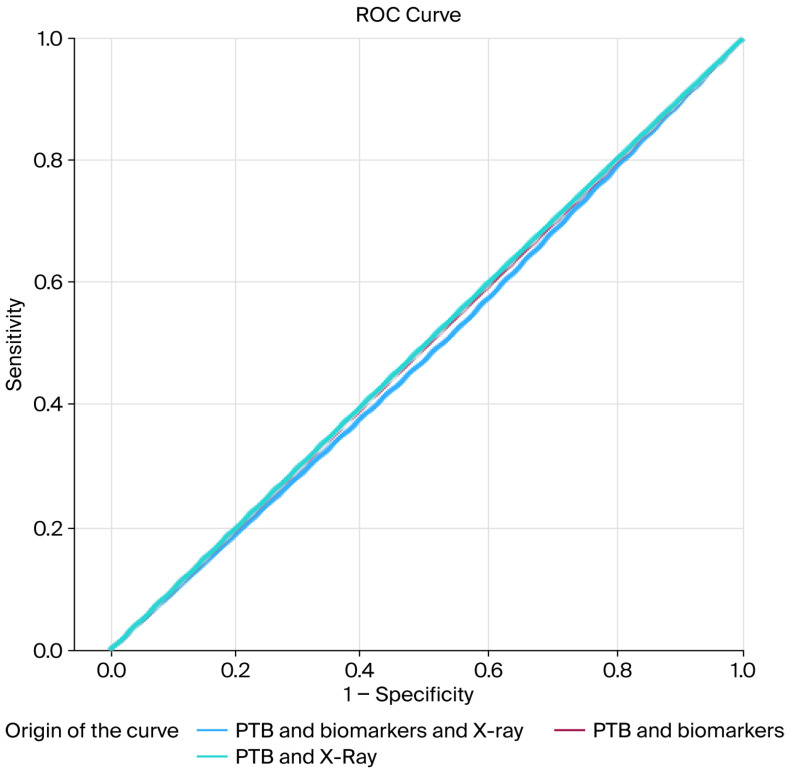
ROC curve of combination of PTB and X-ray; PTB and biomarkers; and PTB, biomarkers, and X-Ray. Abbreviations: X-ray (plain radiography). PTB (probe-to-bone).

Descriptive diagnostic accuracy measures, including sensitivity, specificity, positive predictive value, negative predictive value, and likelihood ratios for the PTB test, plain radiography, biomarkers, and their combinations, are presented in Table 3. Receiver operating characteristic (ROC) curve analyses are shown in Figure 1 and Figure 2.

Overall, the diagnostic tests—both individually and in combination—showed similar performance, with the PTB test demonstrating slightly better diagnostic accuracy compared with the other methods.

The mean duration of ulcer evolution prior to clinical suspicion of diabetic foot osteomyelitis was 23.39 ± 28.29 days. All diagnostic tests were performed at the time of clinical suspicion during the same clinical episode.

## 4. Discussion

The presence of DFO has major clinical implications because it substantially influences treatment outcomes, complication rates, and overall healthcare costs [1,3]. Although bone biopsy remains the gold standard for diagnosis, a combination of clinical assessment and other diagnostic tools—such as the PTB test, plain radiography, and serum biomarkers—has been shown to be useful in routine practice [10,11,24]. Consistent with this, our study demonstrated that the diagnostic performance of PTB combined with blood biomarkers is comparable to that of PTB combined with radiography, suggesting that serum markers may serve as a practical adjunct in the diagnostic evaluation of DFO.

It is important to consider the temporal dynamics of the diagnostic modalities evaluated. Inflammatory biomarkers typically increase early in the course of infection, whereas radiographic changes often appear later, usually after several weeks [25]. In our cohort, all diagnostic tests were performed at the time of clinical suspicion; therefore, differences in diagnostic performance likely reflect the inherent biological behavior of each modality, rather than differences in testing timing. This temporal aspect may partly explain the comparable sensitivity observed between biomarkers and radiography in our study. A further consideration is that the exact onset of osteomyelitis cannot be precisely determined in routine clinical practice; therefore, the duration of suspected infection was estimated based on ulcer evolution.

In our cohort, the PTB test demonstrated a sensitivity of 76% and a specificity of 28%. Grayson et al. first evaluated this test, reporting a sensitivity of 66% and a specificity of 85% [21]. Subsequent studies by Lázaro et al. and Lavery et al. showed even higher diagnostic performance, with sensitivities and specificities of 87% and 91%, respectively [9,14]. Although our specificity values are lower, this discrepancy may be related to differences in population characteristics, ulcer severity, or the high prevalence of neuropathy (93%) in our sample, which may increase the likelihood of a positive PTB test.

Plain radiography demonstrated a sensitivity of 76% in our study. This value is higher than the sensitivity of 54% reported by Álvaro-Afonso et al. [25]. The previous literature has emphasized that accurate interpretation of radiographs requires correlation with clinical findings and detailed knowledge of the ulcer location [26]. Additionally, the diagnostic utility of radiography is limited by its operator dependency and the delayed appearance of radiological changes, which often only emerge in advanced stages of infection.

Serum biomarkers play a key role in the diagnostic assessment of DFO, as diabetic foot infections frequently cause metabolic alterations that influence inflammatory marker levels [27,28]. Numerous studies have highlighted ESR and CRP as the most reliable biochemical indicators of DFO [21,29,30]. Although ESR, CRP, and PCT differ in terms of temporal response and pathophysiological significance, their combined interpretation better reflects routine clinical decision-making. Given that no single biomarker reliably diagnoses diabetic foot osteomyelitis, evaluating inflammatory markers collectively may improve diagnostic sensitivity and reduce false-negative results, particularly in early or atypical presentations [3].

In our cohort, ESR and CRP demonstrated a sensitivity of 74% and a specificity of 20%, consistent with the prior literature. The mean ESR values (32.09 ± 27.81 mm/h) were comparable to those reported by Xu et al., whereas the results for the mean CRP level (11.31 ± 19.10 mg/dL) reinforce its utility as an early-response biomarker [29].

Combined diagnostic strategies did not significantly improve accuracy. PTB plus radiography yielded a sensitivity of 74% and a specificity of 20%, mirroring the performance obtained when PTB was combined with biomarkers. The combination of all three tests also produced similar values. These findings indicate that, in a specialized diabetic foot unit—where clinical expertise is high—the incremental value of adding radiography or biomarkers to the PTB test may be limited. Nevertheless, biomarkers offer two advantages: they provide objective measurements, and they reduce operator-dependent variability, which may be especially useful in settings where radiographic interpretation is less reliable or imaging resources are limited.

It is important to highlight that suspicion of osteomyelitis remains fundamentally clinical. Examination of the ulcer, knowledge of its anatomical location, and assessment of associated signs guide the initial approach to diagnosis. Traditionally, PTB and plain radiography have played a central role in this process [10]. Based on our findings, in cases where radiography is unavailable or where its interpretation is challenging, biomarker evaluation can serve as a complementary tool. Integrating clinical findings with PTB results and serum markers may improve diagnostic consistency across different clinical settings.

An important finding of this study is that the combined use of the three diagnostic modalities—the probe-to-bone test, plain radiography, and serum biomarkers—did not improve diagnostic accuracy compared with the use of two-modality strategies. None of the sensitivity, specificity, and AUC values increased meaningfully when all three tests were combined. This suggests that adding a third modality may not provide incremental diagnostic value and may unnecessarily increase complexity, resource utilization, and the risk of false-positive findings. From a clinical perspective, simpler diagnostic strategies combining two complementary modalities may be sufficient and more practical in routine care.

In our sample, the most frequent ulcer location was the phalanges (25.8%), consistent with international guidelines emphasizing the forefoot as the most common site of DFO. This anatomical distribution underscores the challenge of early detection and highlights the need for accurate diagnostic tools to reduce the risk of amputation and improve patient outcomes.

This study has several important limitations. Firstly, its cross-sectional observational design means that diagnostic tests and reference standards were assessed at a single time point during the same clinical episode, preventing the evaluation of temporal changes in diagnostic performance or the establishment of causal relationships; therefore, prospective longitudinal studies are needed to assess how diagnostic accuracy may evolve over time.

In addition, the sample size was relatively small, particularly among patients with suspected osteomyelitis but negative test results, and all participants were treated in a specialized diabetic foot unit, which may have influenced diagnostic accuracy due to the high level of clinical expertise.

Another relevant limitation is the consistently low specificity observed, indicating a reduced ability of the tests to rule out osteomyelitis when used in isolation, potentially increasing the risk of overdiagnosis. However, in clinical practice, the diagnosis of diabetic foot osteomyelitis is not based solely on complementary diagnostic tests. Careful clinical examination by an experienced clinician remains fundamental and indispensable in accurate diagnosis. When diagnostic test results are interpreted in conjunction with clinical findings and longitudinal wound follow-up, the risk of overdiagnosis can be substantially reduced, thereby avoiding unnecessary antibiotic therapy or other interventions in patients who do not truly require them.

Additionally, each diagnostic modality evaluated in this study presents specific limitations and potential confounding factors that may influence its diagnostic performance. The probe-to-bone test is operator-dependent and may yield false-positive results in patients with deep ulcers, severe neuropathy, or extensive soft tissue involvement, particularly in high-prevalence settings. However, its simplicity and bedside applicability make it a valuable initial screening tool [10].

Plain radiography is limited by its low sensitivity in the early stages of osteomyelitis, as radiographic changes typically appear only after several weeks of infection. In addition, interpretation may be influenced by pre-existing bone abnormalities, prior surgery, or degenerative changes, which can reduce specificity [25,26].

Serum biomarkers are inherently nonspecific and may be influenced by systemic inflammatory conditions, comorbidities, or concurrent infections unrelated to the diabetic foot. Although exclusion criteria were applied to minimize these confounders, biomarker elevation alone cannot reliably distinguish osteomyelitis from soft tissue infection [20].

These limitations highlight that no single modality is sufficient for the diagnosis of diabetic foot osteomyelitis. Diagnostic tests should therefore be interpreted as complementary tools within a comprehensive clinical assessment, integrating physical examination, ulcer characteristics, and longitudinal follow-up to optimize diagnostic accuracy and minimize the risk of misclassification.

Nonetheless, to our knowledge, this is the first study to evaluate the combined diagnostic accuracy of PTB, plain radiography, and serum biomarkers in identifying DFO, and it thus contributes valuable data to inform clinical practice.

## 5. Conclusions

The PTB test combined with serum biomarkers demonstrated diagnostic accuracy comparable to that of PTB combined with plain radiography. This suggests that serum biomarkers represent a practical, accessible, and clinically valuable alternative for diagnosing diabetic foot osteomyelitis, particularly in healthcare settings where imaging resources are limited or radiographic interpretation is challenging. Importantly, the addition of a third diagnostic modality did not improve diagnostic performance. These findings suggest that the use of two complementary diagnostic tools may be sufficient for the evaluation of diabetic foot osteomyelitis, without the need for more complex diagnostic strategies.

## Figures and Tables

**Table 1:** Descriptive analysis.

Variables (N = 128)	Data
Male/female. n (%)	104 (81.3)/24 (18.6)
Diabetes type 1/type 2. n (%)	9 (7)/119 (93)
Age. Mean ± SD	67.27 ± 11.498
BMI. Mean ± SD	30.07 ± 24.55
Time of evolution. Mean ± SD	19.90 ± 13.34
Diabetic neuropathy. n (%)	119 (93)
PAD. n (%)	61 (47.7)
Time of ulcer evolution. Mean ± SD	23.39 ± 28.29
Previous amputation. n (%)	73 (53)
Location of the ulcer. n (%)	
Hallux	22 (17.2)
Phalanges	33 (25.8)
First metatarsal head	12 (9.4)
Central metatarsal head	30 (23.4)
Fifth metatarsal head	15 (10.9)
Hindfoot	4 (3.1)
Midfoot	12 (9.4)
Amputation bed	1 (0.8)
Signs of inflammation n (%)	42 (32.8)
Evolved pathogens. n (%)	
*S. aureus*	28 (21.9)
SCN	11 (8.6)
*S. aeruginosa*	12 (9.4)
*S. epidermidis*	4 (3.1)
*Corynebacterium*	5 (3.9)
*Streptococcus* spp.	4 (3.1)

Abbreviations: BMI (body mass index). PAD (peripheral arterial disease). PTB (probe-to-bone test). ESR (erythrocyte sedimentation rate). CRP (C- reactive protein). PCT (procalcitonin). SD (standard deviation).

**Table 2:** Descriptive analysis of the outcome variables.

Variables (N = 128)	Data
Positive PTB test. n (%)	102 (79.7)
Positive culture. n (%)	94 (73.4)
Positive radiological signs. n (%)	90 (70.3)
Bone marrow	46 (35.9)
Cortical disruption	75 (58.6)
Bone sclerosis	14 (10.9)
New bone formation	3 (2.3)
Sequestrum	6 (4.7)
Involucrum	3 (2.3)
Positive biomarkers. n (%)	107 (83.6)
Elevated ESR. n (%)	65 (50.8)
Elevated CRP. n (%)	90 (70.3)
ESR mean ± SD	32.09 ± 27.81
CRP mean ± SD	11.31 ± 19.10
Elevated PCT. n (%)	9 (7)
PCT mean ± SD	0.18 ± 0.30

Abbreviations: PTB (probe-to-bone test). ESR (erythrocyte sedimentation rate). CRP (C- reactive protein). PCT (procalcitonin). SD (standard deviation).

**Table 3:** Diagnostic accuracy of the test.

	Culture—PTB	Culture—X-Ray	Culture—Biomarkers	Culture—PTB and X-Ray	Culture—PTB and Biomarkers	PTB, X-Ray, and Biomarkers
AUC (*p* value [95% CI])	0.524 (0.702 [0.402–0.646])	0.498 (0.976 [0.377–0.619])	0.483 (0.779 [0.364–0.602])	0.501 (0.981 [0.381–0.622])	0.499 (0.990 [0.378–0.620])	0.488 (0.840 [0.367–0.608])
Sensitivity	0.76	0.76	0.76	0.74	0.75	0.75
Specificity	0.28	0.24	0.23	0.20	0.24	0.23
PPV	0.81	0.72	0.62	0.83	0.69	0.53
NPV	0.23	0.28	0.38	0.13	0.29	0.45
PLR	1.94	0.99	0.99	0.93	0.99	0.96

Abbreviations: PPV (positive predictive value). NPV (negative predictive value). PLR (positive likelihood ratio). NLR (negative likelihood ratio). X-ray (plain radiography). PTB (probe-to-bone test). AUC (area under the curve).

## Data Availability

Data is contained within the article.
